# Older adults' needs and requirements for a comprehensive exergame-based telerehabilitation system: A focus group study

**DOI:** 10.3389/fpubh.2022.1076149

**Published:** 2023-01-11

**Authors:** Julia Seinsche, Eling D. de Bruin, Ilaria Carpinella, Maurizio Ferrarin, Sotiria Moza, Francesco Rizzo, Claudia Salatino, Eleftheria Giannouli

**Affiliations:** ^1^Department of Health Sciences and Technology, Institute of Human Movement Sciences and Sport, Movement Control and Learning, ETH Zurich, Zurich, Switzerland; ^2^Department of Health, OST - Eastern Swiss University of Applied Sciences, St. Gallen, Switzerland; ^3^IRCCS Fondazione Don Carlo Gnocchi Onlus, Milan, Italy; ^4^Materia Group, Nicosia, Cyprus; ^5^Division of Sports and Exercise Medicine, Department of Sport, Exercise and Health, University of Basel, Basel, Switzerland

**Keywords:** older adult, exergame, motor-cognitive training, telerehabilitation, information and communication technologies, qualitative research, User-Centered Design

## Abstract

**Introduction:**

Telerehabilitation in older adults using information and communication technologies (ICTs) provides therapy, which is potentially equally effective as traditional rehabilitation, yet more accessible. This study aimed to analyze the needs and requirements of older adults (OA) and healthcare-professionals (HP) toward ICTs and telerehabilitation in general as well as toward a specific novel exergame-based telerehabilitation system (COCARE system, Dividat).

**Materials and methods:**

The COCARE telerehabilitation system enables individual training based on exergames, as well as an assessment system and a digital centralized case management. Six focus groups with in total 34 participants were conducted. A mixed-methods approach was used comprising questionnaires and semi-structured interviews.

**Results:**

Both OA and HP would engage to an exergame-based telerehabilitation program. Major motivating factors are the relevance of such a training for health and the entertainment component of exergames. Main requirements are simplification of the system, variety, a personalized training, a constantly available contact person, and comprehensive instructions for use. Besides, HP praised the system's motivational effect, but remained concerned about risk of falls and social isolation.

**Conclusion:**

ICTs for telerehabilitation are accepted by OA and HP but should be adapted hardware- and software-wise to address OA' age-stemming vulnerabilities (e.g., risk of falls) and low ICT literacy.

## 1. Introduction

Age-related declines in physical and cognitive functioning and the associated adverse outcomes such as a restricted mobility, cognitive impairment, falls and others ultimately result in a decrease of older adults' (OA) quality of life (1–3). Therefore, the term “active healthy aging” (AHA) increasingly gains in importance in policy frameworks worldwide. Due to a steep growth of the number of people aged 60 years and over (4), there is increased need for long-term care/treatment, which poses a financial challenge for health care systems due to a lack of resources (time and personnel) (1, 5–7). Consequently, conventional rehabilitation often cannot be provided for a sufficiently long period of time to ensure full recovery, which in turn, prevents geriatric patients from reaching their full recovery potential and/or lead an active and healthy lifestyle (8).

Advances in information and communication technologies (ICTs) can present alternative ways of providing health care services as a response to the increased demands on health services (9). As such, ICT-driven advances may play a key role for enabling active and healthy aging as they are being used to support health, wellbeing, and independence of OA (10, 11). These technological advancements enable – among others – telerehabilitation. Telerehabilitation can be defined as the provision of rehabilitation services over distance with the help of ICTs including technology-based training in the home environment as well as a digital centralized remote management (12). Both technology-based training at home environment and remote management proved to be especially important during the COVID-19 pandemic and the imposed social distancing measures prohibiting physical appointments to healthcare professionals (HP) (13). One training approach which efficiently lends itself to telerehabilitation in OA are so-called exergames, i.e., interactive digital games combining motor and cognitive exercises with video games targeting several physical and cognitive functions (14). Previous studies have shown that exergames lead to improvements in several physical functions such as lower extremity muscle strength (15), step reaction time (16), and balance (17–19) as well as in cognitive functions like short-term attentional span (20), processing speed (20, 21), and executive functioning (21, 22).

In the past 10 years, the EU and other funders have devoted billions for ICT-related Research and Development (R&D) projects for AHA. Yet, many products failed to get traction in the market. Thus, to the best of our knowledge, there is no validated, user-friendly geriatric telerehabilitation approach available for AHA that is based on exergames and able to cover the whole continuum of care. One reason for this is that health technology developers often failed to incorporate a user-centered development and design process while developing their products (6, 23). A User-Centered Design (UCD) is an iterative design process that involves all end-users (e.g., patients, caregivers, and healthcare professionals) motivating them to give their opinion about a tool. This involvement is supposed to take place throughout the whole development phases to continuously refine and reshape the design (6) and is, furthermore, recommended by the UK Medical Research Council (MCR) for complex interventions that target to improve health and healthcare (24, 25). So, by applying UCD, a tool's suitability for a specific target group can be assured and the tool's acceptance, functionality, usability and reliability (23) can be optimized. This is of special importance for technological devices designed for OA who express in general less willingness to adopt new technologies in their lives than younger generations. Charness and Boot (26) could show that this reluctance is mainly dependent on attitudes and abilities conflicting with new technologies which are not created for end-users with reduced physical, cognitive, and sensory functions. Therefore, it is crucial to take age-related changes in capabilities into consideration when developing ICTs and to measure requirements and needs of older users toward an ICT-based telerehabilitation system.

Based on the UCD approach, this study aimed to explore the general needs, requirements, and potential barriers of primary (older adults - OA) and secondary (healthcare-professionals - HP) end-users regarding ICTs and telerehabilitation in general, as well specifically regarding a novel exergame-based telerehabilitation system.

## 2. Materials and methods

### 2.1. Materials

**Figure 1:**
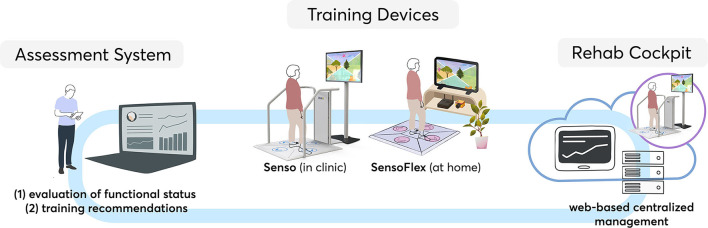
The COCARE-system.

The study was based on the COCARE-system (Dividat, Schindellegi, Switzerland) which provides an exemplary ICT-based telerehabilitation tool for home-based, individual training and therapy as well as a digital centralized case management. It comprises three coupled systems (Figure 1) to enable therapists to provide continuous rehabilitation, remote monitoring, and coaching throughout the whole continuum of care:

(1) Technological hardware devices for motor-cognitive training in clinics (Senso) and at home (Senso Flex)
(2) An assessment system for the analysis of physical and cognitive functioning and training recommendations
(3) A digital web-based management system and rehabilitation cockpit to support the rehabilitation process

**Figure 2:**
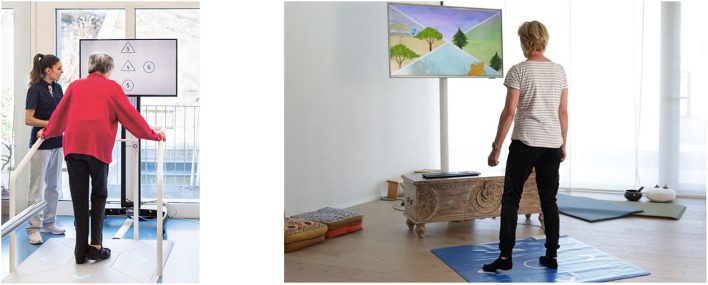
Training in an institution on a Senso and at home on a Senso Flex. Informed consent was obtained from the individuals in the picture allowing the usage of the picture for publication.

The Dividat Senso is a stationary training platform with force sensors, linked to a screen (Figure 2) that delivers fifteen exergames [described in previous studies (16, 17)]. It is already widely used in research (17) and clinical practice (16).

Altogether, the COCARE-system theoretically provides a comprehensive telerehabilitation program comprising assessment, training, and management. However, the Senso Flex (a home-based version of the Senso which consists of a foldable sensor-based mat to be connected to a television/tablet) (Figure 2) as well as the assessment system and the rehabilitation cockpit have only recently been developed, respectively are still in the development phase, and their usability has not been assessed using a recommended (24, 25) iterative approach.

### 2.2. Study design

This was an international, multicenter, cross-sectional study. We used mixed methods to integrate elements of quantitative (questionnaires) and qualitative (focus group interviews) data in three countries (Switzerland (ETH Zurich), Italy (Don Carlo Gnocchi Foundation - FDG) and Cyprus (Materia enterprise)) following Medical Research Council (MRC) guidance (24, 25).

The study design was approved by all local ethics committees (ethics committee of the ETH Zurich (Registration number: 2021-N-104), ethics committee of “IRCCS Fondazione Don Carlo Gnocchi” in Italy (Registration number: 05_09/12/2021) Cyprus National Bioethics Committee (Registration number: EEBK/EΠ/2021/51) and complies with the principles of the Helsinki Declaration.

### 2.3. Recruitment and participants

Participants in Italy were recruited *via* convenience sampling. In Cyprus, OA of existing networks were contacted and cooperations with multifunctional centers for OA were used. Besides, an open invitation to the general public was published. In Switzerland, primary end-users (OA) were recruited through an announcement/advertisement of the University of the Third Age (https://www.seniorenuni.uzh.ch/de.html) of the University of Zurich. Secondary end-users (HP) were recruited in the VAMED Orthopedic Rehabilitation Clinic in Dussnang, Thurgau, Switzerland.

Interested participants were checked for eligibility *via* telephone or in-person. Inclusion criteria for OA were: (1) ≥60 years old, and (2) community-dwelling. Participants were excluded if they (1) were suffering from any severe diseases affecting functional mobility (e.g., severe sensory or motor impairments), which would prevent them from being potential users of the proposed telerehabilitation system, (2) had any diagnosed cognitive impairments that would prevent them from being actively involved in the discussions, and (3) had previous experience with the Dividat Senso. HP had to (1) be actively involved in conducting physical and/or cognitive training with older people as part of their workplace role, and (2) be registered members of the healthcare community. Similar to the OA, HP were excluded if they had previous experience with the Dividat Senso.

Suitable participants who were willing to take part in the focus groups were informed comprehensively about the objectives and the study procedure and signed a written informed consent form. Additionally, all participants gave informed consent that the data collected in this study will be published in a fully anonymized way.

### 2.4. Outcome measures

#### 2.4.1. Semi-structured interviews

Semi-structured interviews were conducted *via* focus groups. Focus groups are the first step of a UCD within the scope of this and other R&D projects. They can be defined as a form of group interview and present a qualitative research method to assemble in-depth knowledge about the ideas, experiences, wishes and requirements of end-users regarding for instance a product or an intervention (6, 27). Each of the three trial sites conducted two focus groups, one with primary end-users (OA) and one with secondary end-users (HP).

#### 2.4.2. Questionnaires

Questionnaires for OA and HP were self-designed to collect socio-demographic data of the participants and, furthermore, to analyze their view on older people's interest in and experience with new technologies and more specifically on a technological home-based rehabilitation program. Items of these questionnaires concerning the participants' individual opinions were evaluated on a 5-point Likert scale (from 1 = ”strongly disagree” to 5 = ”strongly agree”) and each item was analyzed separately, thus, no total score was calculated. Additionally, a few open questions were used to get deeper insight into the participants‘ views. The use of these questionnaires ensured the collection of opinions of less talkative/extroverted persons, who might have been hesitating to actively express their opinions during the focus group interviews. When creating the questionnaires, each question was first formulated in English before being translated into the respective national language of each trial site.

### 2.5. Study procedure

The sessions with OA were planned to last approximately 60 min, whereas the focus groups with HP were more complex and consequently scheduled for about 90 min. The sessions were audio-recorded to ensure the best possible post-processing analysis of the conversations. For all focus groups, a standardized procedure with guiding questions was applied.

After a short introduction of the participants and the interviewer, and explaining the principles of the meeting, participants were asked to fill out the questionnaires assessing experience with and acceptance of technology in general as well as for the use in rehabilitation. Subsequently, the main components of the COCARE-system - Senso and Senso Flex - were demonstrated with a video briefly showing an exemplary application, as well as through a live-demonstration. Basic principles of the assessment system and the rehabilitation cockpit were demonstrated as well. Afterwards, the discussion began with 1–2 general open-ended questions about the participants' attitudes toward new technologies with the purpose to create a relaxed and productive atmosphere. Next, the moderator used pre-defined guiding questions to lead through the main discussion. These guiding questions were specifically product-related, also including business and dissemination matters. Finally, the participants received the opportunity to address possible remaining topics and to draw conclusions.

### 2.6. Data analysis

Verbatim anonymized transcripts were obtained from the audio recordings of each focus group interview. Afterwards, an inductive, qualitative content analysis according to Mayring (28) was performed using the software QCAmap (29). This analysis is based on a structured coding of relevant statements to create a structured overview. The coding was validated *via* inter- and intra-coder agreement. Each item of the questionnaires was analyzed and presented in a descriptive manner.

## 3. Results

**Figure 3:**
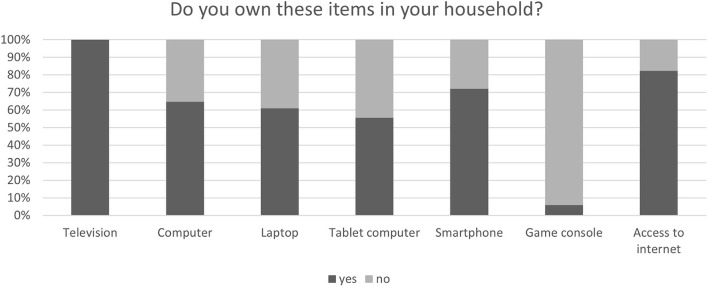
Technologies used at home by older adults.

**Figure 4:**
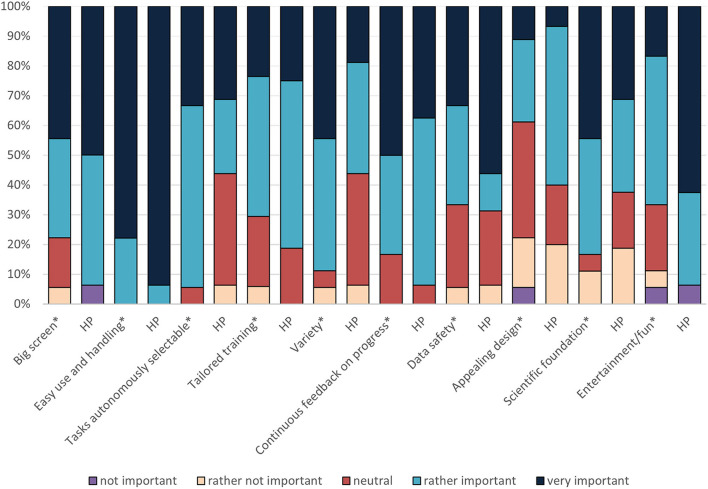
Important factors for a technological home-based rehabilitation program expressed by OA and HP. *Older adults' evaluation; HP, Healthcare Professional.

**Figure 5:**
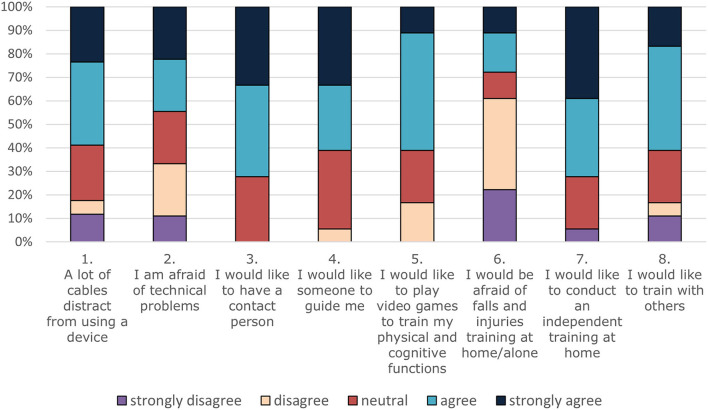
Willingness, concerns, and requirements of older adults toward a home-based rehabilitation program.

In total, six focus groups were conducted (2 focus groups at each site, one with primary and one with secondary end-users) including a total number of 18 OA and 16 HP. Tables 1–**6** and Figures 3–**5** present the summarized results from all three trial sites. In some cases, when site-specific analysis was performed, it is indicated accordingly.

**Table 1:** Characteristics of included OA (*n* = 18).

	**Total**	**Italy**	**Switzerland**	**Cyprus**
Number	18	6	7	5
Sex (female/male)	10/8	1/5	5/2	4/1
Age (in years) (mean ± SD)	76.1 ± 9.7	73.3 ± 8.2	72.0 ± 8.4	85 ± 8.2
years of education (mean ± SD)	12.7 ± 5.1	14.4 ± 2.2	15.8 ± 5,3	8.0 ± 3.7
Sport: times/week (mean ± SD	3.2 ± 0.8	3.0 ± 1.0	3.7 ± 0.5	2.8 ± 1
Sport: hours/week (mean ± SD)	4.9 ± 2.4	5.5 ± 1.3	5.9 ± 3.1	3.3 ± 1.3

OA, Older adults; SD, Standard Deviation.

### 3.1. Demographics OA

In total, OA included in this study had an average age of 76.1 years (SD = 9.7) and the majority described themselves as physically and cognitively fit and healthy not indicating any walking constraints (Tables 1, 2).

**Table 2:** Self-rated health of OA and social factors.

**Health-related and social factors**	**% of all OA (*n* = 18)**
Physically fit and healthy	77.7%
Cognitively fit and healthy	94.4%
Self-reported walking constraints	18.8%
Like to meet new people	100,0%
Social networks user	44,4%

OA, Older adults.

### 3.2. Demographics HP

HP had a mean age of 35.6 years (SD = 13.0) and worked in healthcare 9.5 years (SD = 10.4). Besides, the majority of HP was female (81.3%) (Table 3).

**Table 3:** Characteristics of included HP (*n* = 16).

	**Total**	**Italy**	**Switzerland**	**Cyprus**
Number	16	5	6	5
Sex (female/male)	13/3	4/1	5/1	4/1
Age (in years) (mean ± SD)	35.6 ± 13.0	43 ± 13.5	33.8 ± 15.5	30.4 ± 6.3
Years of work in healthcare (mean ± SD)	9.5 ± 10.4	17 ± 14.2	7.4 ± 8.3	4.4 ± 2.5
Years of work with OA (mean ± SD)	7.6 ± 6.9	12.8 ± 7.3	6.5 ± 7.2	3.8 ± 2.7

HP,Healthcare professionals; SD,Standard Deviation.

### 3.3. Results of the questionnaires—Technological experience and requirements of OA

#### 3.3.1. Technological equipment of OA

Most older participants were well-equipped with different types of technologies in their homes, thus all of them owned a television and about 80% had access to the internet which is a perquisite for using the Senso (Figure 3).

#### 3.3.2. Interest in and experience with new technologies

More than half of OA indicated an interest in (66.6%) and/or a fascination for (70.6%) general modern technologies. Besides, 77.8% expressed a willingness to test new technological devices, although only about 18% indicated having plenty of experience with such devices and although only a small number of OA (16.7%) considered their handling simple. Furthermore, most participants (94.4%) had no experience with video games.

As expected, the majority of OA reported a good basic knowledge and experience with simple operations of common devices like television and a computer/laptop. For instance, about 67% found it easy to turn a computer on and off, to charge a laptop, to navigate through the system and to connect the devices to Wi-Fi. The remaining one third of participants had not tried such operations before. Only connecting the cables of a television or connecting a computer to an external screen posed a challenge for most OA.

More than half of the HP (62.5%) were unsure or tended to be rather pessimistic regarding their perception of older people's interest in new technological devices with only 50% expecting OA to be willing to test them. Table 4 comparing OA's and HP's view on OA's interest in technologies shows that this discrepancy was especially evident in Italy and Switzerland. Accordingly, the vast majority (80%) of all HP was certain that OA would have difficulties handling modern technological devices. Similarly, 56.3% stated that OA have no experience with technological devices and even more (68.8%) that they are unfamiliar with video games.

**Table 4:** Site- and user comparisons of opinions regarding OA's interest in modern technologies.

	**Italy**		**Switzerland**		**Cyprus**	
	**Agree**	**Strongly agree**	**Agree**	**Strongly agree**	**Agree**	**Strongly agree**
OA are interested in new technology (OA/HP)	33/0%	50/20%	29/17%	43/0%	40/80%	0/0%
OA like to test new technological devices (OA/HP)	50/20%	17/20%	43/33%	43/0%	80/80%	0/0%

OA, Older adults; HP, Healthcare Professionals; The statements refer to what OA and HP think about older people's interest in new technologies.

#### 3.3.3. Important factors for an ICT-based rehabilitation program

The following results are based on questions directed more specifically to telerehabilitation. OA and HP were asked to imagine a technological home-based rehabilitation device which is working with video games and allows an independent training at home. Afterwards, they were invited to evaluate the importance of a series of factors for such a training. The results are presented in Figure 4 and Table 5.

**Table 5:** Site- and user-comparisons regarding important factors for home-based rehabilitation.

	**Italy**		**Switzerland**		**Cyprus**	
**Important factors for home-based rehabilitation (OA/HP)**	**Rather important**	**Very important**	**Rather important**	**Very important**	**Rather important**	**Very important**
Big screen	33%/40	67/60%	14/50%	57/50%	20/40%	80/40%
Easy use and handling	50/0%	50/100%	14/0%	86/100%	0/20%	100/80%
Tasks autonomously selectable	50/40%	50/40%	43/0%	43/0%	60/40%	40/60%
Tailored training	40/80%	40/0%	43/50%	0/17%	60/40%	40/60%
Variety	67/40%	33/20%	29/33%	71/0%	40/40%	20/40%
Continuous feedback on progress	50/60%	33/20%	29/67%	57/33%	20/40%	60/60%
Data safety	50/20%	17/60%	14/0%	57/50%	40/20%	20/60%
Appealing design	17/40%	0/20%	57/50%	14/0%	0/75%	20/0%
Scientific foundation	50/40%	50/40%	29/17%	43/33%	40/40%	40/20%
Entertainment and fun	33/40%	0/60%	10017%	0/83%	0/40%	60/40%

OA, Older adults; HP, Healthcare Professionals.

In total, requirements of OA and HP toward such a rehabilitation program resembled one another. Especially the crucial role of an easy use and handling of such devices and continuous feedback on the progress was highlighted by both groups of end-users. However, HP found big screen, an appealing design and entertainment more important than OA did. In contrast, OA rated variety, scientific foundation, and the possibility to select tasks autonomously as more important compared to the HP.

Comparing the two groups of end-users per site (Table 5), the special role of the factors “easy use and handling” and “continuous feedback” was emphasized again, as these factors were rated “rather” or even “very” important by both OA and HP groups of all sites. However, a huge difference between the sites can be detected regarding the importance of the factor “variety” which was essential for all participants in Cyprus (Materia), whereas in Switzerland (ETH) and Italy (FDG) this is only the case for OA and significantly less important for HP. Another interesting finding is that, for all OA, the scientific foundation of the system was a crucial factor – especially in Italy at FDG followed by Cyprus (Materia). At all sites the percentage of HP finding scientific foundation important was about 20% lower. Finally, entertainment was rated as highly important by all HP, whereas only in Switzerland (ETH) OA agreed with HP on this item.

In general, larger discrepancies between views of OA and HP could be observed in Switzerland compared to Italy and Cyprus.

#### 3.3.4. Concerns and requirements of OA toward an ICT-based rehabilitation program

In total, the majority of OA (72%) had a positive view on a home-based training and would like to conduct such a telerehabilitation program (Figure 5, statement 7). Moreover, many participants (61%) expressed a willingness to play video games to increase their physical and cognitive functions (Figure 5, statement 5). However, 58.8% OA were worried about cables distracting them from using the device (Figure 5, statement 1) and even more OA (72.2%) reported that they would need a contact person for support (Figure 5, statement 3). Surprisingly, OA did not report being afraid of technical problems and only few of them (27.8%) indicated having a fear of falls or injuries while training at home.

#### 3.3.5. Training time frame

Table 6 displays how often and for how long OA would be willing to conduct such an ICT-based telerehabilitation program. Most participants (55.6%) would use such a system twice per week and for 30 min (66.7%).

**Table 6:** Training frequency and duration of older adults.

	**Never**	**1x**	**2x**	**3x**	**>3x**
How often per week would you use such a training program?	0.0%	5.6%	55.6%	38.9%	0.0%
	**15 min**	**20 min**	**30 min**	**45 min**	**60 min**
How much time would you invest in one training session?	5.6%	16.7%	66.7%	11.1%	0.0%

### 3.4. Results of the focus group interviews with OA and HP

**Figure 6:**
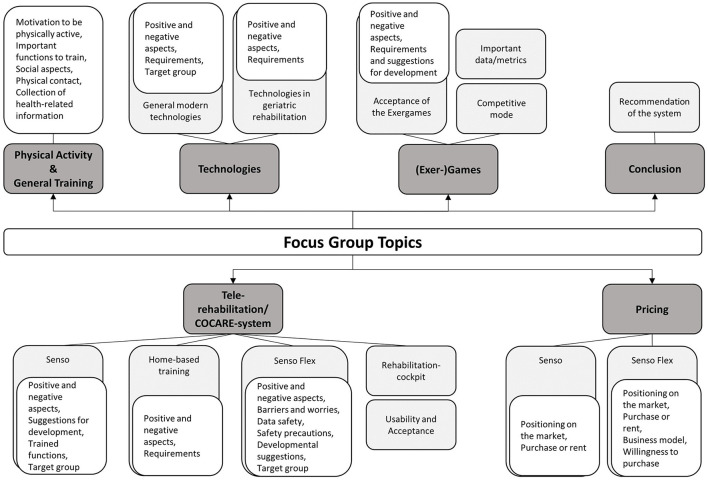
Focus group main- and subcategories. In dark gray: main categories, in light gray: sub-categories level 1, in white: sub-categories level 2.

According to the moderators' observations, participation in the discussion as well as group interaction were good in all focus group interviews. Hence, a large variety of technology- and therapy-related topics arose, creating a comprehensive picture of end-users' needs and requirements toward a technological home-based rehabilitation program. Figure 6 delivers a broad overview over these topics, which are subsequently described in more detail.

#### 3.4.1. Physical activity and general training/therapy

This first main category comprises topics related to general physical activity and exercise. OA mentioned a series of reasons to be physically active, mainly wellbeing and satisfaction, health, and mobility. Fun is apparently a crucial factor to maintain training motivation *(P103: “It has to be a bit of fun, otherwise you won't do it”)*. Moreover, they perceived reaction time, balance, coordination, strength, flexibility, and memory as particularly important functions to be trained.

In general, a major topic arising in both groups of end-users was the importance of social aspects for a successful physiotherapeutic treatment as well as for physical and cognitive training programs. For instance, most end-users agreed on the special importance of guidance, supervision, feedback, and support for effective treatments based on the positive effects of these factors on attention, motivation, adherence, and alignment on shared goals *(P131: “Supervision and feedback from the therapist are important to strengthen motivation and to agree on goals”; P107: the therapist makes sure you're doing it right (…) you can train like crazy, and still do everything wrong”)*. Besides, human presence, and possibly even human touch was said to be particularly important for OA, who, according to HP, tend to be more socially isolated.

#### 3.4.2. General and health-related technologies

Modern technologies and technologies in geriatric rehabilitation emerged as the second main category. Regarding general modern technologies, the views of OA varied. Their main concern was a continuous monitoring by technological devices, and, in addition, some perceived technology as invasive tracking their online activities for commercial purposes. Furthermore, OA tended to regard technologies as an issue more relevant for the younger generations *(P203: “I think, most (technologies) are not meant for people in our age”)*. Nevertheless, alongside this, other OA saw technology as something useful, enjoyable, and nowadays indispensable in all areas of *life (P305: “Without technology we can't do anything. I would say these things are useful”)*. Concerning its purpose in older people's life, they mainly expected technologies to help an older person to remain fit and active, and to be targeted at persons with mobility limitations. Therefore, they would like to see more attention paid to possible physical or cognitive limitations during the development of technologies.

Unlike the opinions on general technologies, OA were generally more open and positive toward technologies specifically designed for the improvement of health. OA recognized their high potential for health-related training programs – especially by providing some form of motivation and feedback to reach a certain goal. Virtual health coaches guiding people toward a healthy lifestyle were mentioned as a good example, as were sleep trackers, pedometers, and heart rate monitors. Nonetheless, some OA remained rather hesitant, mainly due to unfamiliarity with such technologies, while others found them even unnecessary. Some OA, for instance, dismissed technologies like chatbots which were considered demotivating due to the lack of human contact *(P105: “It (the chatbot) was a machine which was talking to me, I could not take it seriously”)*.

Similarly, the majority of HP found technologies useful in geriatric rehabilitation. They recognized, as main advantages, the possibility to extend and intensify treatments while simplifying the therapists‘ work, and the opportunity to increase motivation of the OA *(P111: “I think it's a very good idea (…) I have the feeling that you can attract people better. I've seen it with a MotoMed (a technical, clinical device, which, based on cycling movements, passively or actively trains arm and leg muscles)”)*. However, they regarded good instructions and personalization as prerequisites for the introduction of technologies in rehabilitation *(P109: “Older adults already enjoy technology, but simply do not have the confidence to deal with it. I believe, if you instruct them well, it will help”)*.

#### 3.4.3. Exergames

The third main emerging topic were the Exergames played on Dividat Senso (Flex). In total, OA perceived the exergames positively mentioning positive aspects like a good variety, the adapting algorithm, the trained functions, the game feature/fun aspect and their motivating effect *(P203: “you see it more as a game and less as an exercise”)*. HP shared this positive view, highlighting, in particular, the design, the playful/entertaining aspect which they expect to influence commitment, the variety of games and the possibility to personalize treatments. Moreover, they appreciated the combination of motor and cognitive training/dual-task training *(“The importance of these systems is precisely the combination of motor and cognitive aspects”)*.

Nevertheless, both groups of end-users suggested a series of further developmental steps and constant system updates. OA proposed more age-appropriate games, and the integration of competitive as well as cooperative games. HP, on the other hand, recommended to establish a reward system and wished for the current games to allow for more freedom, such as being able to freely explore a virtual environment while walking on the Senso *(P210: “it would be nice, whenever possible, for the games not to be very restraining”)*.

A final sub-theme within the topic of Exergames was important data/metrics which should be collected and presented during and/or after training with the exergames. HP named speed, accuracy, number of errors, changes over time, number of repetitions, congruence of responses over time, reaction time, and especially progression.

#### 3.4.4. Telerehabilitation and the COCARE-system

Another major topic of the focus group interviews, which included a wide variety of sub-categories, was “telerehabilitation” and “the COCARE system.” Starting with the system‘s main device (i.e., Senso), most OA expressed positive views about it. For instance, many of them praised the feedback on performance and the possibility to track improvements over time *(P201: “It gives more accurate results (than a therapist) (…) and places the patient at a more central point of the overall process”)*. However, some participants named limited movements as a major critical aspect *(P308: “(…) limited in terms of movements … to move only between the four arrows”)*. The HP's focus remained on the handrail of the Senso. On the one hand, it was associated with a feeling of security, but on the other hand, they feared that older people might seek unnecessary support limiting the training effects on balance. Other therapists suggested that a solution supporting people with standing difficulties would be a meaningful addition to the Senso. Moreover, many HP as well as some OA would endorse the integration of upper body movements and, finally, the further development of the assessment system to detect initial stages of motor and cognitive deterioration.

Concerning the physical and cognitive functions trained on the Senso, most HP found that the following are well-targeted: balance, postural stability, attention, concentration, reaction, and visual-motor integration. However, they would wish for memory and endurance to be addressed more extensively through the exergames. Several HP criticized that, compared to the cognitive demand, the challenge on the motor system remains underdosed. Likewise, OA regarded reactivity as one of the major functions trained on the Senso but indicated the lack of a higher physical demand *(P304: “It seems particularly suitable for improving reaction times (…) by increasing the speed of movements, perception (….) when someone walks on the street (…) he/she is more reactive to potential risks”)*. Consequently, according to the OA, people suffering from mobility limitations could represent an appropriate target group for a Senso training, whereas currently they perceive themselves as too fit to be included in the target group *(P106: “We probably feel it's still relatively easy now … but maybe in 10–15 years it won't be”)*.

The second important sub-category within the topic of telerehabilitation is general home-based training. Concerning OA, the number of positive and negative opinions was well-balanced. The most frequently named positive aspect was the possibility to regularly conduct exercises despite possible mobility limitations which currently became even more significant due to the COVID-19 pandemic. Moreover, even the older participants were aware of a possible relief of hospitals due to home-based training. Two negative aspects from the OA' point of view were, however, the risk of falls during training alone, and concerns that especially older people might not want to stay at home in front of the television for physical, social, and psychological reasons – a fear which is shared by HP. Besides, older participants would miss immediate feedback from the therapist. HP saw clear advantages in home-based training as, according to them, training in a familiar environment leads to more wellbeing meanwhile. Furthermore, time and flexibility were presented as crucial arguments - especially during the winter months when leaving the houses poses a higher risk for falls *(P111: “You can use it at any time – it is flexible in terms of time”)*. Nevertheless, they feared for their patients' adherence due to their lack of control. Eventually, both groups of end-users regarded a therapy plan similar to usual treatments and regular feedback by therapists as perquisites for a successful home-based rehabilitation program *(P309: “(…) patient periodically receives feedback from the therapist on how the home rehabilitation process is proceeding”)*.

The third telerehabilitation related topic was home-based training specifically conducted with the Senso Flex. Most end-users' positive as well as negative views on the Senso Flex were based on their opinions about general home-based training and exergames listed above. Examples of such overlapping statements by OA are the special importance of Senso Flex for people with mobility limitations but also its negative effect on social connectivity. Senso Flex specific positive ratings mainly comprised its small size, the fact that it can provide a diversion in everyday life, and that it can be used for general exercise *(P202: “It could be used not only for rehabilitation but also in terms of general exercising to stay fit”)*. Senso Flex specific negative evaluations included storage difficulties and space problems. Data safety, however, was not regarded as a major concern. Concerning the view of HP, overall, the Senso Flex received positive evaluations. For instance, they praised the clearly visible markings on the carpet, its thinness, its provision of diversion in everyday life and in time spent with friends for entertainment purposes, and finally the possibility to use it not only for prevention and rehabilitation but also for general *fitness (P111: “You can perhaps do something other than only having the usual coffee with colleagues who are visiting. So, I see advantages here, too”)*. Still, according to HP, further necessary steps comprise the development of suitable presentations of training data. In addition, a worry both groups of end-users shared were safety issues due to balance problems as well as technological unfamiliarity. For this reason, it was emphasized that therapists would have a responsibility to figure out individual safety precautions *(P109: “The therapist co-decides or advises where the mat should be put to ensure that the environment is safe”)*. Finally, the term “simplification” repeatedly arose in all focus groups referring to both installation and use of the Senso Flex meaning adaptions like a vocal introduction and feedback and the most possible reduction of buttons and cables *(P107: “It should be very easy to handle so that you can start exercising after just a few actions”)*. Nevertheless, concerning familiarization with the Senso Flex, a significant majority of participants was optimistic provided that an appropriate guidance or even installation by the therapist as well as a constant contact person are available. Furthermore, both groups of end-users emphasized that a familiarization should already begin in clinics during rehabilitation starting with a supervised training on the Senso before transferring to the Senso Flex *(P108: “we (HP) can also initiate as much as possible from our side, so that they (OA) have as little effort as possible”)*.

However, regardless of such adaptions or familiarization efforts, many participants would prefer using the COCARE-system as an extension rather than a substitute for usual therapy. Besides, they regard patients in late rehabilitation as the most appropriate target group of the Senso Flex, whereas there was a general conviction that people with cognitive disorders will be incapable of using the system.

In summary, according to the vast majority of OA and HP, usability and acceptance of the Senso Flex are strongly dependent on its ease of use, good instructions and feedback, older people's physical and cognitive abilities, and enjoyment.

Concerning the final sub-category, the rehab-cockpit, healthcare-professionals highly approved its inclusion – however, only in case personal meetings would still take place. Both groups of end-users would, furthermore, appreciate a reward system as well as reminders and recommendations regarding the choice of game to play.

#### 3.4.5. Pricing

The final category, pricing, included topics like positioning on the market, purchase or rent and the participants willingness to purchase the system. Concerning the price, it was difficult for most end-users to estimate a suitable price and consensus could not be reached. However, for most end-users, renting the Senso or Senso Flex would be the preferred model due to financial reasons and difficulties to estimate how long one will be able and willing to use the system *(P203: “It depends on how many years you have to live (…) the older you are the less likely it would be to buy it”)*. A general concern prevailed, that OA might not be able to afford the system in any way, which is why many participants expected Health Insurance Companies or National Health System to cover the costs.

It can be concluded that the willingness to pay for the Senso Flex is strongly dependent on the costs, effectiveness, and the ability and will to independently use it *(P201: “I would buy it if I was convinced it worked and could improve my health. Especially if my physiotherapists would recommend it”)*.

## 4. Discussion

In general, the use of ICTs for telehealth has proven to reduce health care costs, improve self-monitoring of health and enhance the provision of rehabilitation programs to OA (30). Thereby, exergames can be a useful and effective part of an ICT-based telerehabilitation tool as they have proven to be very effective due to the simultaneous conduction of physical and cognitive exercises – a combination which, according to previous research, may be even more effective than conducting them separately (31) or in more traditional exercise programs (32). Thus, exergames enable the training and improvement of a large variety of physical functions (33) such as balance (17–19), aspects of gait, gait initiation (34), dual task walking speed (16), and movement quality (35) as well as cognitive and psychological functions like executive control and processing speed (21), exercise enjoyment (36), decreased depressive symptoms, and an increased mental health-related quality of life (37).

However, negative attitudes based on fear, anxiety and limited motivation and interest form a barrier for the adoption of ICTs by OA (38). This again illustrates the importance of a UCD-approach applied during the development of the COCARE (and any) system to identify the factors necessary for a successful implementation. Indeed, all focus groups delivered a comprehensive picture illustrating needs, requirements, and potential barriers of OA and HP toward the COCARE-system. Therefore, it can be concluded that the study objectives have been achieved.

### 4.1. Opinions and requirements toward ICTs and the COCARE-system

When specifically discussing the COCARE-system, a term which was repeatedly stated was “simplification,” i.e., an easy use and handling, which was mentioned as a prerequisite to reach a good usability and acceptance among OA and HP. This is well in line with previous research (39) which stated that a successful implementation and adoption of technologies depends on perceived costs, for instance cognitive costs and self-efficacy beliefs (40). Technologies which are difficult to handle increase cognitive costs, decrease self-efficacy beliefs and consequently the willingness to use ICTs. To overcome these obstacles, other essential demands by all end-users were the availability of a contact person, good instructions, a personalized therapy plan, and regular as well as immediate feedback concerning training conduction, progression, and recommendations. Summarizing all these factors, it becomes evident that a good education about ICTs and guidance are crucial for the older participants' acceptance of technologies. This is in accordance with previous studies highlighting the special importance of education on how to use new technologies in order to dismantle fears of OA due to unfamiliarity with ICTs and to instead change their attitude toward ICTs (41–43), and increase their wellbeing and confidence in handling them (30, 44–46). Thereby, the focus should be on changing attitudes and self-efficacy beliefs which have proven to have a greater effect on older people's use of ICTs than actual knowledge (40, 47). This points to the necessity of a blended therapy approach where conventional face-to-face care is combined with telerehabilitation (TR) (48, 49). Future iterations within the development process should shed a light on this.

However, next to targeting the perceived costs and technological unfamiliarity, (perceived) benefits are also important. The fact that the older participants' view on technologies for rehabilitation was more positive than their view on general technologies indicates that personal benefits and a meaningful purpose of technologies play a key role for their adoption of technology. This is in line with previous research (50).

Concerning safety aspects, at the beginning, OA indicated no fear of falling, whereas later in the interviews they criticized the risk of falls when using the Senso Flex. An explanation for this seeming discrepancy might be, that in the first case, they were specifically asked about their own fears, whereas in the second case they might also had other frailer OA end-users in mind. HP were more consistent requiring several safety precautions like handrails for the independent use of the Senso Flex.

### 4.2. Exergames

All end-users had a positive view on the exergames provided by the Senso – especially due to their entertaining nature leading to a higher motivation, their variety, and the cognitive and physical functions they train. Besides, the possibility to track improvements was repeatedly praised. Enjoyment and health were factors also listed as very important motivators to be physically active which explains these positive responses. This is mirrored in previous research where enjoyment was a key motivator for playing exergames (51).

Based on these positive views on the exergames, we expect increased chances of high training adherence in exergame training. This indeed could be shown by previous research which attributed this mainly to a high enjoyment (51). However, another study (52) observed a decrease in adherence in exergame training compared to conventional training which, according to the authors, could be explained by the low level of social interaction – an assumption supported by further studies (51, 53). Similarly, in the current study, a large majority of both groups of end-users feared a lack of social contacts when training with the Senso Flex. This factor turned out to be a very decisive and a major reason for many OA and HP to agree to the idea of the COCARE-system as a supplement to conventional therapy rather than a replacement. To counteract the loss of social contact, Oesch et al. (52) proposed exercising with others and collecting points which could enhance motivation and adherence. This is in accordance with the opinion of OA who required the integration of cooperative and competitive games – an aspect which should be considered when designing future exergames. Another point to consider when designing ICTs is that communication should remain as much as possible on a personal level instead of replacing the therapists' feedback with a chatbot or similar technologies.

Moreover, some OA criticized the imbalance between the cognitive and physical demands of the games. However, it must be taken into consideration that the older participant's perceived themselves as physically healthy. Besides, on average, they appeared to be well-informed about various aspects of physical and cognitive functions which became evident when they presented detailed ideas and high expectations of what an effective training should include, respectively which functions it should aim at. This was also demonstrated when they rated “scientific foundation” as a highly important factor for a telerehabilitation program – even more important than “entertainment.” This was somewhat contrasting the view of HP that did not deem an evidence-base as likewise important. Therefore, the development and scientific evaluation of further games to establish a solid evidence-base for effectiveness seems crucial to win physically healthy and well-educated OA as primary end-users. Especially games targeting a higher level of endurance and strength, or the integration of upper body movements should be considered.

### 4.3. Potential users

Despite this, in general, there were disagreements and uncertainties in both groups regarding the appropriate target group of the Senso Flex. As indicated above, according to many participants, the COCARE-system's main users will be patients in late rehabilitation, however, some also recognized its potential as a tool for prevention, offering, in addition, a welcomed diversion in everyday life. In contrast, people with cognitive limitations were disregarded as potential users which is not in line with previous research showing exergames as well as telerehabilitation are feasible and effective treatment approaches for people with mild cognitive impairment (MCI) (54–56) or dementia (57–59). However, the combination of telerehabilitation and exergames in older people with cognitive impairment has not yet been analyzed sufficiently. Thus, the participants' doubts should not be ignored and, consequently, a definition of the COCARE system's target group(s) is necessary before working on further developments.

### 4.4. Differences between groups of end-users and sites

As listed above, HP most often agree with OA regarding important needs and requirements – especially regarding social factors and guidance. Besides, they agree that OA have limited experience and abilities regarding new technologies. However, it became evident that HP tended to underestimate older people's interest in and experience with technology. This is most likely based on ageism which is even occurring among physiotherapists (60). Furthermore, another explanation might lie in their occupation which regularly confronts them with older people having cognitive and/or physical disabilities, whereas the older participants of the current study were, as described, on average rather healthy. So, in fact, most OA would be willing to conduct such a home-based training with technological devices.

Furthermore, surprisingly, HP did not assign similarly great importance to the factor “scientific foundation,” whereas they rate “entertainment” significantly higher. Previous research (61–64) investigated physiotherapists' reasons for their selection of treatment methods and found that “most reported interventions are supported by evidence, interventions with unclear or no evidence of effect were also used to a high extent.” (61). Instead, they relied on their initial education, other therapists, and on gained experience. This could also be regarded as another explanation for their pessimism toward older people's interest in exergame-based training as this most likely was not part of their education. Moreover, it could explain the site differences. Among the healthcare-professionals in Switzerland the number of young professionals was higher than in Italy at FDG or in Cyprus at Materia and, therefore, they were even more influenced by their education. This might explain why they found “scientific foundation” the least important compared to therapists of the other two sites since evidence-based physiotherapy implementation is associated with many barriers by physiotherapists (65). Eventually, this indicates a need to integrate the theory of ICTs for the use in physiotherapeutic treatments in the curriculum of therapists.

Accordingly, the fact that HP regarded entertainment as a much more important factor might be based on their experience teaching them that enjoyment is vital for the adherence of OA to their therapy. This again might be a reason why HP mentioned more freedom in exergames as another important developmental step, whereby OA focused more on adaptations with respect to age and physical and cognitive demands.

### 4.5. Limitations

Although this study provides important insights about needs and requirements of OA toward healthcare technologies in general and especially toward the COCARE system, it is important to acknowledge some limitations. Due to organization issues and COVID-19 restrictions, only few of the focus groups‘ participants tried out the Senso and Senso Flex and only a small selection of games could be shown. The rest of the participants stood close by and observed. This might have caused a limited reliability of their evaluation of difficulty of the games. Furthermore, as described above, it must be considered that the older participants were physically and cognitively in good condition compared to peers and also their exposure to and use of technologies is higher than the average in this age group. This is confirmed by previous surveys: according to Eurostat and the Swiss Federal Office for Statistics, in 2021, about 61% of OA in EU (Cyprus 58%, Italy 45%) (66), and about 73% in Switzerland used the internet - especially for sending emails (67), whereas the percentage of OA regularly using the internet was significantly higher (82%) in our study. One explanation for this finding might be their high level of education (47). In any case, these differences lead to a reduced representativeness of the study sample. However, the numbers of older adults being familiar with the internet and technologies is increasing (68) and likewise the generalizability of the presented results.

## 5. Conclusions

Unlike the predictions of HP, the OA showed an interest in technologies for the improvement of health and in using the COCARE system as a tool for telerehabilitation. However, some adaptions like a simplified installation of and navigation through the system were required. Furthermore, the importance of social factors was strongly emphasized by both groups of end-users. Based on these results, the COCARE-system is about to be adapted and, as a next step within the UCD-approach, the adapted version will be investigated again analyzing it's usability and acceptability. The information gained in this study is part of an iterative approach to develop a complex health intervention (24) and warrants further iterative cycles of development with stakeholder input and system adaptations.

## Data availability statement

The raw data supporting the conclusions of this article will be made available by the authors, without undue reservation.

## Ethics statement

The studies involving human participants were reviewed and approved by Ethics Committee of the ETH Zurich. The patients/participants provided their written informed consent to participate in this study.

## Author contributions

JS: conceptualization, methodology, data curation, software, formal analysis, and writing—original draft. EG: conceptualization, methodology, supervision, and writing—original draft. EB: methodology. IC, MF, SM, FR, and CS: methodology, data curation, and writing—review and editing. All authors revised the manuscript and approved the version submitted for publication and agree to be accountable for all aspects of the work.
